# A Review of the Potential Effects of Melatonin in Compromised Mitochondrial Redox Activities in Elderly Patients With COVID-19

**DOI:** 10.3389/fnut.2022.865321

**Published:** 2022-06-20

**Authors:** Wen-Lin Su, Chia-Chao Wu, Shu-Fang Vivienne Wu, Mei-Chen Lee, Min-Tser Liao, Kuo-Cheng Lu, Chien-Lin Lu

**Affiliations:** ^1^Division of Pulmonary and Critical Care Medicine, Department of Internal Medicine, Taipei Tzu Chi Hospital, Buddhist Tzu Chi Medical Foundation, New Taipei City, Taiwan; ^2^School of Medicine, Tzu Chi University, Hualien, Taiwan; ^3^Division of Nephrology, Department of Internal Medicine, Tri-Service General Hospital, National Defense Medical Center, Taipei, Taiwan; ^4^Department and Graduate Institute of Microbiology and Immunology, National Defense Medical Center, Taipei, Taiwan; ^5^School of Nursing, National Taipei University of Nursing and Health Sciences, Taipei, Taiwan; ^6^Department of Pediatrics, Taoyuan Armed Forces General Hospital Hsinchu Branch, Hsinchu City, Taiwan; ^7^Department of Pediatrics, Tri-Service General Hospital, National Defense Medical Center, Taipei, Taiwan; ^8^Division of Nephrology, Department of Medicine, Taipei Tzu Chi Hospital, Buddhist Tzu Chi Medical Foundation, New Taipei City, Taiwan; ^9^Division of Nephrology, Department of Medicine, Fu Jen Catholic University Hospital, School of Medicine, Fu Jen Catholic University, New Taipei City, Taiwan

**Keywords:** COVID-19, melatonin, mitochondria, mitophagy, oxidative stress, SARS-CoV-2

## Abstract

Melatonin, an endogenous indoleamine, is an antioxidant and anti-inflammatory molecule widely distributed in the body. It efficiently regulates pro-inflammatory and anti-inflammatory cytokines under various pathophysiological conditions. The melatonin rhythm, which is strongly associated with oxidative lesions and mitochondrial dysfunction, is also observed during the biological process of aging. Melatonin levels decline considerably with age and are related to numerous age-related illnesses. The signs of aging, including immune aging, increased basal inflammation, mitochondrial dysfunction, significant telomeric abrasion, and disrupted autophagy, contribute to the increased severity of severe acute respiratory syndrome coronavirus 2 (SARS-CoV-2) infection. These characteristics can worsen the pathophysiological response of the elderly to SARS-CoV-2 and pose an additional risk of accelerating biological aging even after recovery. This review explains that the death rate of coronavirus disease (COVID-19) increases with chronic diseases and age, and the decline in melatonin levels, which is closely related to the mitochondrial dysfunction in the patient, affects the virus-related death rate. Further, melatonin can enhance mitochondrial function and limit virus-related diseases. Hence, melatonin supplementation in older people may be beneficial for the treatment of COVID-19.

## Introduction

The new severe acute respiratory syndrome coronavirus 2 (SARS-CoV-2) is responsible for causing coronavirus disease (COVID-19). Regardless of the patient’s age, SARS-CoV-2 increases the clinical morbidity and mortality of individuals with underlying chronic diseases or poor immunity. This is especially true for the elderly. Although the virulence of SARS-CoV-2 is high, pathogen–host interactions result in significant differences in the severity of the illness, its complications in humans, and survival outcomes. Previous studies have proposed that components of aging, which are interactive with COVID-19, can affect the health of older people through mechanisms that regulate the immune system (1, 2).

Aging is a complex and multifactorial process (3). As cells age, their ability to cope with external and internal stress and their functionality gradually diminish, whereas disease morbidity and mortality gradually increase. Age-related immune system degeneration, known as immune senescence, increases the sensitivity of the elderly to infectious diseases, autoimmune diseases, and cancer. As a part of the normal physiological function for fighting pathogens, the mitochondria generate low levels of reactive oxygen species (ROS) (4). However, excessive ROS production can be as damaging as coronavirus infection (5). Many viruses also induce mtROS production, which can aid viral replication and the release of progeny (6). The increase in ROS production is related to age-related diseases and a reduced lifespan (7). Furthermore, the ROS detoxification system significantly weakens with age. A reverse correlation between glutathione peroxidase (GSH-Px) and age has also been found (8). Aging damages the mitochondrial electron transport chain (ETC). The induction of stress in the endoplasmic reticulum (ER) damages the ETC in human mitochondria, which are in close contact with the ER. ER stress gradually increases with age and leads to mitochondrial dysfunction. Hence, the reduction of ER stress is a potential strategy for enhancing the function of the cardiac mitochondria in the elderly (9). Autophagy is also an important protein degradation process in normal cellular metabolism. Therefore, the reduction in autophagy owing to aging can aggravate the severity and prognosis of COVID-19 in elderly patients, since worsening age-related autophagy accelerates viral infections by reducing virus degradation and mitigating innate and adaptive immunity (10).

Impaired cellular respiration reduces ATP production, increases ROS production, decreases cell detoxification, and impairs autophagy and immune dysfunction, which seem to play critical roles in the inflammatory state and disease severity in elderly patients with COVID-19. Melatonin, associated with almost all aging-related processes, is primarily released from the pineal gland and regulates the circadian rhythm. The retina, lacrimal glands, Harderian gland, gastrointestinal tract, thrombocytes, and bone marrow are other sources of melatonin (11). Thus, melatonin is a widespread physiological mediator (12). It is also an effective free radical scavenger that influences the immune system considerably (13). However, melatonin synthesis and its levels in the pineal gland and plasma gradually decrease with aging. In animals, treatment with exogenous melatonin may postpone aging and the onset of age-related illnesses (14).

The review primarily focuses on how melatonin can decrease the severity or accelerate the recovery of older adults with COVID-19 by protecting damaged mitochondria.

## The Relationship of SARS-CoV-2 with Aging, Immune System, And Oxidative Stress

It is common knowledge that asymptomatic patients with COVID-19 can increase the risk of virus spread. This poses a serious threat to older people, who may suffer from severe complications owing to virus–host interactions (15), including sudden stroke or cardiovascular events (16). Hence, awareness of the adaptability of the immune response to SARS-CoV-2 infections can lead to better clinical care and management for older people (2, 17).

During aging, the basal metabolism and physiological function largely depend on the mitochondria. The mitochondria are the major source of ATP and are crucial for various processes in cell survival, such as β-oxidation of fatty acids, calcium signaling, generation of ROS, phospholipid biosynthesis, and apoptosis (18). Intracellular mitochondria are the main sites for oxygen consumption and ROS production; therefore, they are the prime targets of ROS-dependent cell damage. Mitochondrial respiration can also affect a cell’s longevity by modifying the ROS equilibrium (19). Aging is characterized by the increased production of ROS. However, despite the current interest in antioxidant supplementation, such as CoQ10 supplementation, there is insufficient evidence to recommend CoQ10 supplementation as an anti-aging and antioxidant therapy (20). However, CoQ10 supplementation improves respiratory viral infections and the critical inflammatory status (21). With age, the mitochondrial phosphorus capacity and ATP production decline. Hence, the significant increase in energy consumption due to a cytokine storm establishes a non-adaptive state in which the metabolic reserve of the mitochondria significantly increases in elderly patients with COVID-19.

### COVID-19 and Aging

**FIGURE 1:**
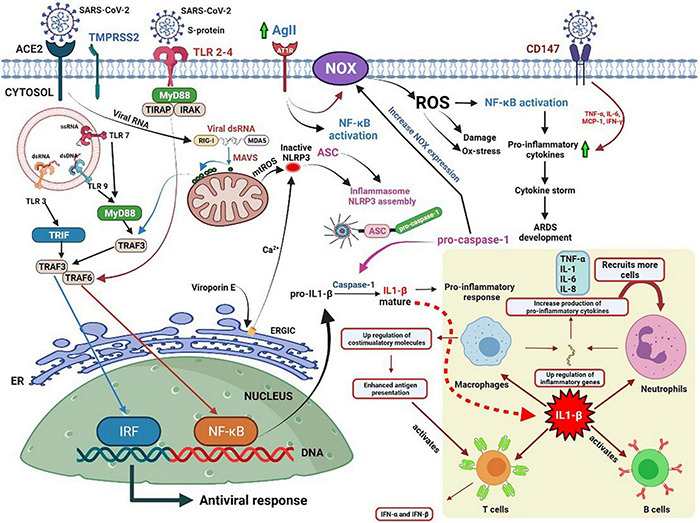
Potential pathways of oxidative stress and the role of IL-1β in eliciting immune responses against coronavirus disease.

Normal senescence involves the degeneration of cells, tissues, and organs, which increases mortality and morbidity in the elderly. The various aspects of aging include immune aging, inflammation and inflammasome formation, mitochondrial dysfunction, oxidative stress, telomere shortening, and impaired autophagy. Such changes cause the decline of various reserves and adaptability, all aspects of system function, in addition to poor response to the mutual influence of pressure of infection and interference with cells. The various aspects of aging also play a critical role in patients with chronic diseases (22, 23) and may affect SARS-CoV-2 infection (Figure 1). Hence, with the COVID-19 pandemic still at large, it is necessary to consider these characteristics when treating infected elderly patients (24).

SARS-CoV-2 binds to the angiotensin-converting enzyme 2 (ACE2) receptor and penetrates the host cell through endocytosis. Foreign nucleic acids from the invading viruses are detected by multiple intracellular pattern recognition receptors (PRRs). The PRRs include DNA and RNA receptors located in the cytoplasm as well as specific Toll-like receptors (TLRs) expressed in endolysosomal compartments. The coronavirus spike protein is recognized by TLR2/4, and the single-stranded RNA (ssRNA) is recognized by TLR7/8. Additionally, double-stranded RNA (dsRNA), which exists as an RNA virus replication intermediary, is recognized by RIG-I/melanoma differentiation-associated protein 5 (MDA5). The detection of dsRNA leads to coordination with mitochondrial outer membrane antiviral signal protein (MAVS) followed by oligomerization, attracting a variety of adaptor proteins and ultimately activating nuclear factor kappa light chain enhancer of activated B cells (NF-κB) and interferon regulatory factors (IRFs). TLR detection leads to the recruitment of adaptor proteins, which can form myddosomes to facilitate the activation of NF-κB. NF-κB signaling is closely associated with the expression of IL precursors. The activation of the nucleotide-binding oligomerization domain-like receptor (NLR) family pyrin domain-containing 3 (NLRP3) inflammasome facilitates the oligomerization and recruitment of apoptosis-associated speck-like proteins with a caspase-recruitment domain. The resultant inflammasome transforms pro-caspase-1 into active caspase-1 for further processing of pro-ILs.

Further, the stimulation of NF-κB signaling is related to an increase in NADPH oxidase (NOX) expression, which can also be induced by angiotensin II (AGII), followed by the production of ROS. However, ROS itself can induce NF-κB signal transduction. Therefore, excessive ROS production mediates excessive inflammation and the induction of cytokine storms, which might lead to the development of acute respiratory distress syndrome (ARDS) and worsen the severity of the disease (16).

The bottom right corner of Figure 1 illustrates the role of IL-1 in initiating an immune response to infection. IL-1 enhances the extensive pro-inflammatory activity of various immune cells. It not only induces the rapid recruitment of neutrophils to the site of infection, the activation of endothelial adhesion molecules, and the induction of chemokines, but also promotes the release of various cytokines. Furthermore, it induces the proliferation of T helper cells and B lymphocytes and improves the antigen presentation capability of APCs (25).

Viroporin E, an integral ion membrane channel protein of SARS-CoV-2, can modify the ionic flux of Ca^2+^, K^+^, and H^+^ in the ER, which induces ROS production, autophagy, and ER stress, and drives NLRP3 activation. IL-1β induces the expression of adhesion molecules and integrins on leukocytes, endothelial cells, and other cell types, promoting cell infiltration and inflammation. IL-1 regulates neutrophil recruitment by inducing the expression of adhesion molecules (ICAM-1 and β2 integrins) and the production of local chemokines in the endothelium. It upregulates the production of IL-8, which acts as a chemotactic and activating factor for neutrophils, endothelial cells, and macrophages. SARS-CoV-2 infection enhances the expression of AGII, which activates NOX through the AGII type 1 receptor, and in turn, triggers the production of ROS and subsequent damage (26). SARS-CoV-2 may recognize the CD147 receptor (a glycoprotein that can cause a cytokine storm in lung epithelial cells) on the surface of the host cell by binding the viral nucleocapsid protein to the CD147 ligand cyclophilin A (27). However, studies have shown that melatonin can reduce tissue damage by blocking the production of pro-inflammatory cytokines associated with CD147 activity in patients with COVID-19 (28).

### Innate and Adaptive Immunosenescence, Inflammation, and Inflammasomes

**FIGURE 2:**
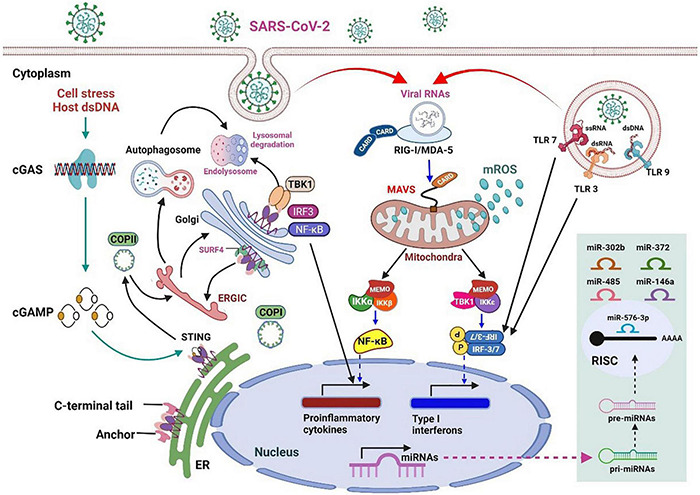
An overview of innate immunity against RNA virus infection and the stress response to host cell infection in mammals.

A prominent structural protein of SARS-CoV-2 is the spike glycoprotein (S), which contains two major subunits, S1 and S2. Other coronavirus proteins include the membrane protein (M) from the host ER or Golgi apparatus, mainly responsible for virus assembly; the nucleocapsid (N), involved in genome replication; and the envelope protein (E). SARS-CoV-2 is similar to SARS-CoV-1, and the S-protein amino acids of both the viruses are nearly 80% similar (29). Furthermore, the concordance of N, M, and 3a proteins in SARS-CoV-1 and SARS-CoV-2 suggests that they share the same pathogenicity (Figure 2).

RNA virus infection in mammalian cells is first detected by host RRPs (RIG-I and MDA5), which initiates the retinoic acid–inducible gene-I (RIG-I)-like receptor (RLR) signaling pathway. MAVS acts as a linker for PRRs, which then activates NF-κB or IRF3/7, leading to the rapid and effective production of pro-inflammatory cytokines and IFN-I (early). During this process, the mitochondria act as a signaling platform on the outer membrane to activate various downstream molecules. The dynamic mitochondrial properties under the mitochondrial outer membrane (MOM), such as inner membrane potential (Δψm), oxidative phosphorylation activity, and mitochondria-related metabolites, also help fine-tune signaling events. The topological gap between mitochondria-related metabolites and MAVS is filled with the prohibitin complex, which establishes a bridge between the MOM and the mitochondrial inner membrane (MIM) when a virus infects (30). Approximately 24 h after infection (late stage), some miRNAs are significantly upregulated (Figure 2, light green box on the lower right), which mediate various regulatory immune responses. miRNAs, such as miR-146a and miR-576-3p, negatively regulate the antiviral response after viral infection, which is essential to terminate RLR over-signaling.

In addition, the mitochondria are involved in the cyclic GMP-AMP synthase (cGAS)-stimulator of interferon genes (STING) signaling pathway, activated by viral infection-induced host cell stress and dsDNA (31). The schematic on the left in Figure 2 details the activation of cGAS induced by the dsDNA of the host cell owing to pathogenic infection or host cellular stress. cGAS dimers assemble upon binding to dsDNA, leading to the activation of the cGAS enzyme and the synthesis of 2′,3′ cyclic GMP–AMP (cGAMP). cGAMP binding to the STING dimer located on the ER membrane significantly changes the conformation, triggers STING oligomerization, induces release from anchoring factors such as stromal interaction molecule 1, and, along with the transport factor, gets incorporated into the coat protein complex II vesicles. While passing through the ERGIC and Golgi apparatus, STING not only recruits the TNF receptor-associated factor (TRAF) family member-associated NF-κB activator-binding kinase 1 (TBK1) and IRF3 but also promotes the phosphorylation of TBK1 and Ser366 of STING. The phosphorylation of IRF3 by TBK1 leads to the dimerization and translocation of IRF3 to the nucleus to induce the expression of IFN1, interferon-stimulated genes (ISGs), and genes encoding several other inflammatory mediators, pro-apoptotic enzymes, and chemokines. The activation of STING also activates NF-κB and the formation of light chain (LC)3+ vesicles (autophagosomes). Finally, STING in the autophagosome and those from the Golgi apparatus to the lysosome are involved in STING degradation (32).

Animal and human cells mainly use PRRs to recognize pathogen-associated molecular patterns (PAMPs) and endogenous danger-associated molecular patterns (DAMPs). There are various prominent PRRs, such as TLR, NLR, and cytoplasmic RLR, which are essential regulators of inflammatory and innate immune responses. Viral ssRNA is primarily recognized by TLR, such as TLR-7 (33), which subsequently stimulates the production of pro-inflammatory cytokines and type I and III IFNs (34). The first step in limiting viral entry or replication is the synthesis of IFNs and their release from virus-infected cells, which upregulates ISGs (35). IFNs can boost the activity of macrophages, natural killer (NK) cells, and T and B lymphocytes to inhibit virus assembly, spread, and disturbances in the immune system (36). Some studies have suggested that the coronavirus may not only antagonize the production of IFNs but also reduce the transmission of IFN signals to escape immune surveillance (37) (Figure 1).

The RIG-I receptor, present on the MOM, can recognize viral RNA and activate MAVS proteins. However, these proteins can also increase pro-inflammatory cytokine production by activating the NF-κB signaling pathway (38). The overall increase in baseline inflammation during general aging contributes to advanced age-related chronic diseases (39). Furthermore, viral infection can exacerbate age-related immune response injury by stimulating the inflammatory pathways. With aging, decreased innate immunity against viral infections weakens IFN synthesis from neutrophils, monocytes, macrophages, and NK cells and their subsequent response to IFN signaling (40, 41). In addition, costimulatory signals are attenuated by different APCs, which activate T cells in advanced age (42). Hence, old age leads to functional changes in these immune cells, which can result in deviant innate immune responses.

The NLRP3 inflammasome is an oligomeric complex that may promote the secretion of IL-1β and IL-18 and induce pyroptosis by defending the host from microorganisms. It has been demonstrated that coronaviruses vitalize the NLRP3 inflammasome and the NF-κB pathway (43), resulting in increased levels of TNF-α conversion enzyme (TACE), TNF receptors, and ACE2. ACE2 enhances the production of angiotensin 1–7 (44) (Figure 1). An earlier report revealed that ACE2 and TACE levels are associated with poor prognosis in patients with heart disease (45). However, ACE2 expression during human aging and its activity following SARS-CoV-2 infection remain unclear. COVID-19 has led us to focus on the vulnerability of aging populations to emerging diseases. This predisposition to illness and death is also a major challenge in the development of vaccines and immunotherapeutics. The efficacy of vaccines decreases significantly with age because of the gradual age-related decline in innate and adaptive immunity (46).

The adaptive immune response targets non-self-pathogens, but it may occasionally attack host cells erroneously. Adaptive immunity includes humoral and cellular immunization, which identify and respond to specific pathogens *via* B cells and T cells, respectively. After infection, B lymphocytes are activated, differentiated into plasma cells, and produce immunoglobulins. This property can be induced by cytokines generated by CD4^+^ cells (47). As people age, there is a significant shift from naïve to memory B cells and a decline in the antigen recognition capacity and antibody production. Moreover, the long-lived plasmocyte population is reduced in response to vaccination (48). The number of naïve T cells and the synergy between T-cells and APCs, which can transform naïve T cells into memory cells, are reduced (49). Notably, an age-related gender gap has been observed in the immune system, with a higher incidence of COVID-19 in men than in women (50). The epigenetic and transcriptomic variance associated with age-dependent immune cell dysfunction may also underlie the age-related differences in the severity of COVID-19 symptoms (51).

### Genomic Instability and Telomere Attrition

The accumulation of mutations in various tissues and cell types in aging humans can lead to the age-related breakdown and death of cells. Since the somatic mutation rate is considerably higher than the germline mutation rate, and the base replacement load in somatic cells is adequately high, it may affect the function of immune cells (52). By extending the cell cycle, p53 allows additional time for the repair machinery to restore genome stability and plays a leading role as a promoter of DNA repair. In the context of DNA repair mechanisms, p53 lowers stress levels and protects cells from oxidative lesions (53). In contrast, higher levels of oxidative stress (such as viral infection) will cause the continuous activation of p53 and increase the permeability of the MOM, which increases the release of cytochrome c, ultimately causing apoptosis (1, 54). Although p53 can reduce the replication of coronaviruses by prolonging the cell cycle, papain-like protease (PLpro) produced by the coronavirus itself can degrade p53, enabling the virus to continue replicating in infected cells (55, 56). Many viruses damage the DNA of host cells, resulting in genetic instability throughout their replication cycle. DNA viruses activate and manipulate the DNA damage response. Even if they only replicate in the cytoplasm, many RNA viruses can cause severe DNA damage in host cells. DNA damage can increase the risk of RNA virus pathogenicity by triggering apoptosis, stimulating inflammatory immune responses, and introducing harmful mutations that increase tumor risk (57). Notably, some SARS-CoV accessory proteins regulate the IFN signaling pathway and the production of pro-inflammatory cytokines. For example, the coronavirus accessory protein 7a can mediate subsequent cell apoptosis by inhibiting Bcl-X_*L*_ (an anti-apoptotic protein) (58).

Telomeres (repetitive TTAGGG DNA sequence at the end of linear chromosomes) form a part of the 3D spatial organization of the nuclear genome. They stabilize the genome with high fidelity throughout early adulthood, but their effects weaken after the reproductive age (59). They also play a crucial role in maintaining the stability of the genome and regulating innate immunity while combatting viral infections. Regions close to telomeres, namely the sub-telomeres, contain GC-enriched genes that regulate innate immunity (60). Varying lengths of telomeres (61) are present in various aging cells, which may be the basis of different severities of responses to viral attacks (60). During aging, a decrease in telomerase activity occurs with the gradual shortening of telomeres; shorter telomeres are associated with more severe diseases (62). Previous studies have shown that the telomere/telomerase system can alleviate the harmful effects of aging on lymphocytes, which may help identify high-risk individuals (63). Thus, viral infection can cause telomere attrition, resulting in serious clinical consequences for the elderly with COVID-19, and telomere attrition may guide future treatments against SARS-CoV-2 and other viral pathogens.

## SARS-CoV-2 and the Mitochondria

Under normal physiological conditions, the mitochondria play pivotal roles in metabolic oxidation (through the tricarboxylic acid cycle), ATP production (through an efficient ETC), and complete β-oxidation of fatty acids. In mammals, the mitochondria are also involved in the innate immune response against various microorganisms or toxic environmental stimuli (31). The mitochondria produce ROS at low levels, which may aid immune cell maturation and function; however, at high levels, ROS may increase oxidative damage (4). All components of the mitochondrial ETC, including complexes I, II, and III, produce ROS in the matrix or inner membrane, and mtROS may regulate NF-κB- and TNF-α-mediated cell death and aid cellular transformation. Many signaling pathways, such as hypoxia and PI3-kinase-induced mtROS production, control mtROS production, and FOXOs regulate mtROS production under oxidative stress (64). Importantly, some viral proteins may interact with the mitochondrial ETC and produce ROS, which may promote virus replication and release (6). Therefore, mtROS overproduction may be enhanced upon virus infection and aid replication to promote the survival of the virus.

The mitochondria also regulate innate and adaptive immunity, which stimulate NF-κB, the NLRP3 pathways, and IRFs. Mitochondria-mediated innate immunity is induced by activating the signaling pathways of NLRP3 inflammasomes and RLRs. MAVS and the mitochondrial components are critical for signal transduction (31). Mitochondrial DNA (mtDNA) can be regarded as a DAMP and serves as a crucial platform for signaling molecules, such as MAVS, in RIG-I signal transduction and NLRP3 inflammasomes (65).

Mitochondrial biogenesis, fusion, and fission play a role in activating immune cells. In addition, the mitochondria mediate cytotoxic responses to cellular stress (65). The combination of respiratory failure, decreased ATP production and detoxification capacity, increased ROS production, and immune dysfunction appears to contribute to the onset of fulminant inflammation and severe COVID-19 (66).

### Chaperone–Protease Networks

**FIGURE 3:**
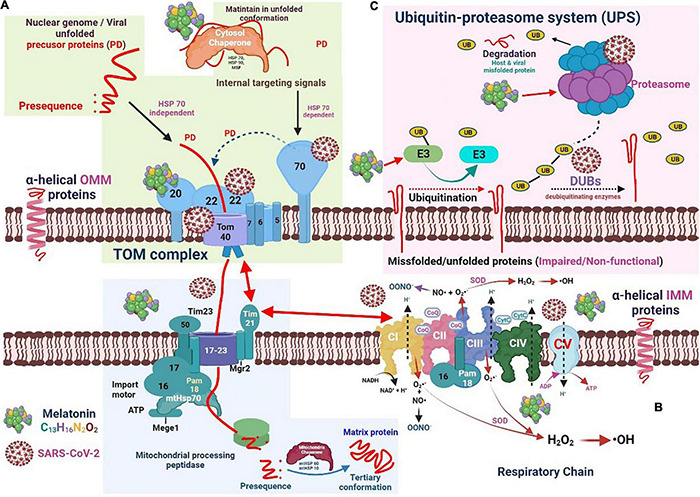
The interaction of severe acute respiratory syndrome coronavirus 2 and melatonin with the chaperone-protease system and the respiratory electron transport chain machinery. **(A)** Viral unfolded precursor proteins translocate across mitochondrial membranes into the intermembrane space or mitochondrial matrix. Melatonin may regulate HSPs and proteasome activity which can improve mitochondrial function and restrict viral replication. **(B)** The mitochondrial electron transport complex (CI-CV) involved in oxidative phosphorylation, generating ATP. Free radicals are generated when electrons leak and reduce adjacent oxygen molecules to superoxide anion radicals (O_2_^•−^). The pyrrole ring in melatonin may be suitable for capturing O_2_^•−^ and ^•^OH free radicals. **(C)** In the mitochondrial outer membrane, the ubiquitin-proteasome system (UPS) drives the quality control of proteins. Melatonin may decrease the level of downregulated protein 4-1 (E3 ligase).

Most mitochondrial proteins are produced in the cytoplasmic ribosomes and pass through one or two mitochondrial membranes into the intermembrane space or mitochondrial matrix (67). These proteins are unfolded, which allows them to effectively translocate across the mitochondrial membrane. Translocations catalyze these highly dynamic machines induced by potential membrane reactions, ATP, or redox reactions. They coordinate with chaperones and assembly complexes to steer mitochondrial proteins to the correct position. Translocation is carried out through tight pores formed by closely synchronized translocases, including the translocases of the outer and inner membrane complexes (TOM and TIM, respectively) (Figure 3A) (68). Adequate mitochondrial protein homeostasis is based on endogenous enzyme components comprising a diverse set of chaperones and proteases forming an interconnected functional network (69).

SARS-CoV-2 uses the normal protein translocation mechanism of the mitochondria to suppress the antiviral cellular immune response and effectively maintain viral replication. SARS-CoV-2 can interact with TOM components to prevent the synergistic effect between Tom70 and MAVS, suppressing the activity of mitochondrial IRF3 and subsequently reducing the induction of antiviral signal response (70). SARS-CoV polyproteins exhibit a deubiquitinating activity that attenuates the ubiquitination capacity of infected cells. The SARS-CoV nucleocapsid protein can interact with the proteasome and suppress its proteolytic activity, contributing to the inhibition of viral protein proteolysis and suppression of host misfolded protein degradation (71). Figure 3B illustrates the relative positions of the mitochondrial electron transport complex (CI–CV), which forms the ETC involved in oxidative phosphorylation, ultimately generating energy in the form of ATP. When electrons leak and reduce adjacent oxygen molecules to superoxide anion radicals (O_2_^•^-), free radicals are generated. CIII secretes electrons into the matrix and inner membrane space, whereas CI secretes electrons into the mitochondrial matrix. In addition, superoxide dismutase (SOD) can transform O_2_^•^- into hydrogen peroxide by converting it to hydroxyl radicals (^•^OH). O_2_^•^- can also combine with nitric oxide to generate peroxynitrite anions.

Under normal conditions, the mitochondrial precursor protein exposes the hydrophobic area, leading to unwanted protein interactions and aggregation. Thus, before TOM translocation, these unfolded precursor proteins must bind to a cytoplasmic chaperone to enter the mitochondria (72). TOM is a transport protein containing the surface receptors that recognize pre-proteins and is the main entry point of the mitochondria. Mitochondrial protein biogenesis usually involves complex folding and assembly processes for the attainment of enzymatically active states. Molecular chaperone proteins, such as heat shock protein (Hsp) 70, promote the translocation and folding of mitochondrial proteins (69). The chaperone activity of heat shock cognate protein 70 (Hsc70), Hsp70, and Hsp90 is derived from the collaborative cycle of ATP hydrolysis and substrate binding and is regulated by numerous accessory chaperone proteins. These chaperones transfer the precursor protein to the Tom70 receptor by recognizing the signal embedded in the membrane-targeted precursor (73). Mitochondrial precursor proteins can be attached by an Hsp70-dependent (Tom20 and Tom70) or Hsp70-independent (Tom20 only) manner and then pass through the Tom40 tunnel (74). At this point, the cytosolic chaperon mitochondrial import stimulant factor binds with Tom70 and transfers the precursor protein to Tom20 and Tom22, which uses energy from ATP hydrolysis (74). When transferred through TOM, the pre-sequence protein is directed toward the Tim23 complex, essential for importing cleavable pre-proteins into the mitochondria. Tim23 (the Tim21 and Mgr2 subunits) is involved in the interaction between the TOM and respiratory chain complex. Pre-proteins contain amino-terminal targeting sequences removed through mitochondrial processing peptidase and/or internal membrane protease (IMP). The cytosolic precursor of Mgr2 contains a carboxy-terminal sequence that promotes mitochondrial targeting, whereas Mgr2 is treated by IMP (75).

The presequence translocase-associated motor (PAM) transports precursor proteins into the mitochondrial matrix, mostly in an ATP-dependent manner. Tim44 is a subunit protein in the mitochondrial matrix that can bind to the Tim17–Tim23 complex core. In addition, Tim44 interacts with mitochondrial heat shock 70 kDa protein (mtHsp70) and recruits chaperones and their associated proteins into the translocation channel (76). Pam18 and Pam16 subunits form a stable heterodimer, which may stimulate the ATPase activity of mtHsp70 and are connected to multiple import channels (77). Hydrophobic proteins use the Hsp70/90 complex and TOMM34 to prevent misfolding in the cytoplasm. Once the protein enters the mitochondria, the mitochondrial chaperone protein system (composed of Hsp10 and Hsp60) bends the polypeptide chain in a precise tertiary configuration (78).

In the MOM, the ubiquitin-proteasome system (UPS) drives the quality control of proteins (Figure 3C). In general, when conventional methods cannot bend proteins into their natural configuration, these proteins are targeted by the UPS for degradation (79). The UPS complex can efficiently eliminate damaged and/or malfunctioning proteins. Under normal circumstances, the UPS and autophagy-lysosomal pathway are two important mechanisms for the repair or removal of abnormal proteins. The UPS is responsible for eliminating mitochondrial proteins, whereas the autophagy-lysosome pathway is responsible for the degradation of the entire organelle by lysosomes (80).

Foreign and native proteins are digested into small peptides, triggering an adaptive immune response. In eukaryotic cells, the proteasome is responsible for almost all ATP-dependent proteolytic processes. Ubiquitin is a protein with 76 amino acids, and its binding to the substrate protein residues (monoubiquitination) is responsible for the degradation of small proteins mediated by the proteasome. The polyubiquitin chain is constructed by linking each subsequent ubiquitin molecule to the lysine residue present in the preceding ubiquitin (81). Ubiquitination is reversed by the deubiquitinating enzyme (DUB), which removes or alters the ubiquitin chain to counteract the E3 ligase activity (82). Alterations in proteasome activity lead to mitochondrial dysfunction. Additionally, different components of the UPS and DUB are present in the mitochondria simultaneously. These findings show the close relationship between mitochondrial proteins and the UPS. As the mitochondria are the major source of ROS, they may also be damaged by oxidation. The UPS uses other quality control mechanisms to eliminate damaged mitochondrial proteins. Excessive ROS can oxidize and destroy proteasome subunits, thereby reducing their catalytic activity (83).

### Utilization of the Chaperone–Protease Complex System by SARS-CoV-2

The virus can generate various components and virions by activating the host cell machinery. Viruses use ongoing scalable strategies to interfere with infected cells and create an environment conducive to their replication and survival (84). MAVS is a central regulator of antiviral and inflammatory responses (85), and viruses have developed strategies to suppress the antiviral response of host MAVS signaling. Viruses can also damage mitochondrial reproduction by altering their dynamic morphology (86). In addition, a host of viral proteins can be found in the mitochondria during viral replication, where they can interact with mitochondrial proteins and manipulate various mitochondrial function and components, such as oxidative homeostasis maintenance, MPTPs, Δψm, electron transport, and ATP production (84).

**FIGURE 4:**
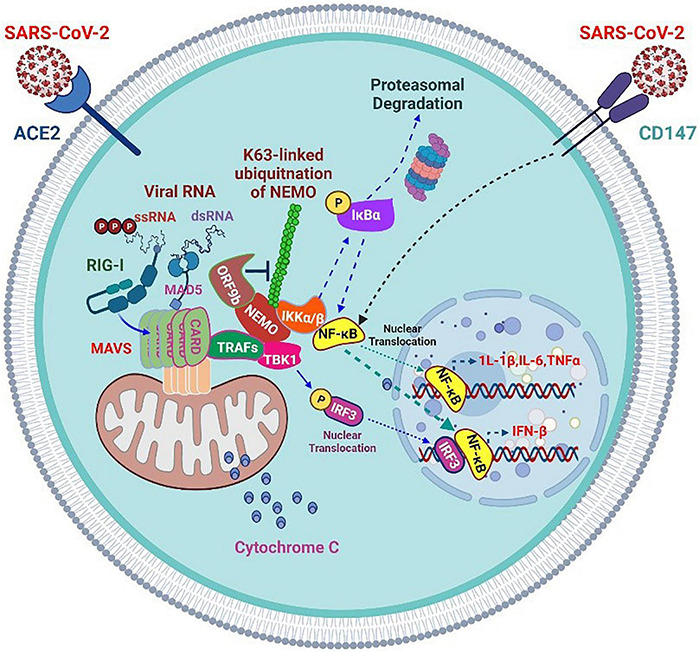
Severe acute respiratory syndrome coronavirus 2 accessory proteins inhibit RIG-I/MAD5-MAVS antiviral signaling but enhance CD147 inflammatory signaling.

Various proteins are synthesized in a relatively short time during viral replication. Therefore, adequate protein folding cannot be performed in a timely and effective manner. Consequently, most viruses require chaperones throughout their life cycle for the proper folding of proteins (Figure 3). For instance, the Hsp70 levels elevate after viral infection, which is critical for virus proliferation. In addition to issues related to protein folding, viruses interfere with cellular signal transduction, cell cycle regulation, and apoptosis induction, creating an environment favorable for their proliferation and preventing/delaying the premature death of host cells (87). SARS-CoV-2 ORF9b suppresses the IFN-1 response by targeting Tom70 (88). Intracellular RNA viruses are detected by RIG-I/MDA5, which triggers the formation of the MAVS signaling complex in the mitochondria. When infecting cells forming MAVS, Tom70 interacts with RNA viruses and induces antiviral responses by enhancing IRF3-mediated gene expression (70). Therefore, joining SARS-CoV-2 ORF9b with Tom70 suppresses the IFN-I response by disrupting the MAVS signal (88). RNA-Seq profiling studies of paired ribosomes demonstrated that SARS-CoV-2 non-structural protein (NSP) 1 inhibits the translation of Tom22 and Tom40 in host cells, consequently altering the mitochondrial import of precursor proteins (88). Further, the SARS-CoV-2 NSP4 protein interacts with components of the TIM complex and affects the import of MIM proteins (89). The ORF9b protein can interfere with the nuclear translocation of NF-κB or p65 by disrupting the polyubiquitination of K63-related NF-κB essential regulators [inhibitors of NF-κB kinase subunit (IKK) γ], thereby inhibiting the RIG -I-MAVS signal and weakening the IFN-I response (90) (Figure 4).

The SARS-CoV-2 infection progresses mostly in two stages. Studies have confirmed significant immunosuppression in the early stages of COVID-19 (91). Most patients with mild or asymptomatic COVID-19 demonstrate mild immunosuppression, which may promote the shedding and spread of a large number of viruses. ORF9b and other SARS-CoV-2 proteins (such as NSP13, NSP14, NSP15, and ORF6) are effective IFN antagonists in these early stages. For example, SARS-CoV-2 uses the early protein ORF9b to mask antiviral defense and inflammatory responses in newly infected patients.

However, when COVID-19 progresses to later stages, most patients with the severe disease develop ARDS with a highly inflammatory state. Numerous pro-inflammatory cytokines are produced at this time point, and the lymphocyte population is reduced. The levels of IL-6, IL-10, and C-reactive protein increase substantially, which is a reliable indicator of severe COVID-19 (92, 93). Although the inflammatory response is vigorous, the production of IFN in patients at advanced stages of the disease is hindered. The delayed IFN expression and unbalanced host response may be partly attributed to impaired IRF3 signal transduction, which is also the target of other SARS-CoV-2 accessory proteins, such as ORF6. Previous studies have confirmed that SARS-CoV-2 NSP1, NSP3, NSP12, NSP13, NSP14, ORF3, ORF6, and M proteins may inhibit the virus-induced activation of the IFN-β promoter. In-depth analysis shows that ORF6 not only inhibits the production of type I IFN but also reduces the downstream signal transduction of IFN itself (94). SARS-CoV-2 ORF6 has been confirmed to exert the most obvious inhibitory effect on primary IFN production and the IFN signaling pathway (95).

SARS-CoV-2 ORF9c is a 73-amino acid protein that interacts with membrane proteins from several cellular organelles and alters antiviral processes. It targets the path of IFN through interaction with negative MAVS controllers (NLRX1 and NDFIP2) (96). SARS-CoV-2 NSP13, NSP14, NSP15, and ORF6 are strong IFN antagonists (95) (Figure 4). NSP13 targets the trajectory of IFN by interacting with the MAVS TBK1 effector (89). The SARS-CoV-2 M glycoprotein impairs the aggregation of MAVS and its recruitment of downstream signaling components, such as TRAF3, TBK1, and IRF3, resulting in the inhibition of downstream IFN-1 antiviral genes. In addition, it inhibits the stimulatory effects of RIG-I, MDA5, MAVS, and TBK1 on the IFN-I promoter (97). Furthermore, it inhibits the production of IFN-I by preventing the formation of a functional complex containing TRAF3, mediated by its first transmembrane domain (TM1). Mechanistically, TM1 is capable of binding with RIG-I, TRAF3, TBK1, and IKKε and prevents the interaction of TRAF3 with its downstream effectors (98).

In addition to effectively removing incorrectly folded proteins from cells, the ubiquitin-proteasome (UPS) system also acts as a host defense mechanism for removing viral components. Viruses can disrupt or manipulate the cellular mechanism underlying the functioning of the UPS to promote their reproduction and block the host immune response. In some cases, viruses encode proteins with E3 activity or even form a part of the E3 cell complex to degrade cellular proteins that limit the spread of the virus (99). The coronavirus genes include two open-reading frames (ORFs), ORFs 1a and 1b, which produce two viral polyproteins, pp1a and pp1ab, respectively, which are further cleaved by viral proteases to produce functional NSPs (100). Two coronavirus proteases, papain-like protease (PLpro; released from NSP3) and 3C protease (3CLpro), are especially critical for coronavirus replication (100). The 3CLpro processes the C-terminus of the viral polyproteins pp1a and pp1ab, whereas PLpro processes its N-terminus. In addition, SARS-CoV PLpro exerts a deubiquitinating effects on host cell proteins (101), which inhibits the breakdown of poorly folded viral and host proteins. Moreover, the nucleocapsid protein of SARS-CoV interacts with the p42 proteasome subunit, resulting in altered viral proteolysis and escape of SARS-CoV from immune surveillance (102, 103). The inhibition of the proteolytic activity of proteases may also affect the degradation of poorly folded host proteins.

In docking analysis conducted in drug research, the binding mode of some proposed compounds with the SARS-CoV-2 PLpro protein has been used to establish a basic intermolecular interaction spectrum. Comparative analysis with known standard inhibitors also indicates that these proposed compounds may serve as potential active chemical entities that regulate the SARS-CoV-2 PLpro protein. Therefore, targeting PLpro may be a crucial therapeutic strategy for COVID-19 (104).

### Effect of Melatonin in the Mitochondrial Chaperone-Protease Complex System

Melatonin can affect the molecular chaperone–protease network by regulating the expression of HSPs, Tom20, and Tim23, and interacting with the mechanism of action of the UPS (105, 106). In cold stress-induced immunosuppression studies, exogenous melatonin treatment not only improved cold-induced immunosuppression but also upregulated the expression of HSF-1 and HSP-70 in immune cells, which prevented protein unfolding and cell death (107). The increase in the expression of HSPs is beneficial for promoting the anti-apoptotic effect and sirtuin-1 (SIRT1) expression and suppressing the activation of inflammatory p38 MAPK and NF-κB pathways (108). Melatonin exhibited neuroprotective effects by reducing oxidative stress and HSP70 expression in ovariectomized rats with chronic brain hypoperfusion (109). The overexpression of HSP70, induced by changes in antioxidant defense, is considered to exert a protective effect by directly preventing inflammation and pathways of cell destruction, such as apoptosis and necrosis. However, melatonin can reverse the increased oxidative stress-induced HSP70 expression by suppressing the altered antioxidant status (109). In cardiomyocyte hypoxia experiments, melatonin improved mitochondrial metabolism, reduced excessive mitochondrial oxidative stress, induced effective mitochondrial fusion, and prevented mitochondrial apoptosis. Further, melatonin can enhance the expression of Tim23 and Tom20, resulting in the enhanced expression of adenosine monophosphate (AMP)-activated protein kinases, such as 5′ AMP-activated protein kinase (AMPK) and peroxisome proliferator-activated receptor-γ coactivator 1α (PGC-1α) (110).

In hematological malignancies, melatonin can inhibit the proteasome, which is closely related to the regulation of major signal transduction proteins, including p53, NF-κB, caspase-3/9, apoptosis factors BAX and BIM, and anti-apoptotic factors BCL-2, TRAIL, nuclear factor erythroid 2-related factor 2 (NRF2), and β-catenin (111). Melatonin also regulates various cellular functions by interacting with the UPS. In pathological hypoxia with high proteasome levels, melatonin decreases the level of downregulated protein 4-1 (E3 ligase in neuronal cells) expressed by neural precursor cells, thereby weakening proteasome activity. However, melatonin does not directly affect UPS (111). Direct evidence shows that melatonin inhibits proteasomes in human kidney cancer cells. Additionally, melatonin therapy induces apoptosis and regulates the expression of the pro-apoptotic protein BCL-2-mediating cell death (BIM) in renal cancer cells (112).

In aging animal models, melatonin may enhance protease activity to support cellular health. Aging exerts a significant negative effect on proteasome activity in various tissues, which leads to the increased vulnerability of cells to oxidative stress and inflammation. This is because the inhibition of the protein clearance mechanism is directly related to aging. Under these circumstances, melatonin may inhibit the buildup of abnormal proteins in cells by increasing proteasome activity (105). During aging, circadian disturbances cause a decrease in melatonin production, which is also linked to reduced proteasome viability (106). The regulatory effect of melatonin on HSPs and proteasome activity can improve mitochondrial function and restrict the replication of SARS-CoV-2 (Figure 3A). Because COVID-19 is more severe in older patients, and melatonin and proteasome levels gradually decrease with age, melatonin supplementation may be helpful in older patients undergoing COVID-19 treatment.

## Effects of SARS-CoV-2 on Mitochondrial Dynamics and Autophagy

Autophagy is used by cells to eliminate invasive pathogens, such as SARS-CoV-2. Autophagy is involved in several critical functions of protein complexes. In general, the mammalian rapamycin complex 1 (mTORC1)/unc-51-like autophagy activating kinase (ULK)1/2 complex inhibits autophagy in the absence of stress. Stress, including that resulting from viral infections, inhibits mTORC1, which subsequently activates a series of proteins, leading to autophagy and viral particle packaging and degradation (113). Autophagy also regulates the link between the innate and adaptive immune responses to viral infections by inducing antigen-presenting cells (APCs, such as macrophages). APCs present viral antigens to CD4+ T cells to release cytokines and subsequently regulate adaptive immune responses. In early autophagy, CD4^+^ T cells are induced to release interferon gamma (IFN-γ), which promotes the response of CD8^+^ T cells, NK cells, and macrophages in the elimination of virus particles (114, 115). In addition, autophagy can moderate the inflammatory response by promoting IL-1β expression for lysosomal degradation to prevent the inflammatory pathway and ROS accumulation (116). Even though some viruses may escape surveillance related to autophagy, autophagy-related immune effects can still be used to combat viral infection and prevent inflammation-induced organ injury.

### Mitochondrial Fission–Fusion

**FIGURE 5:**
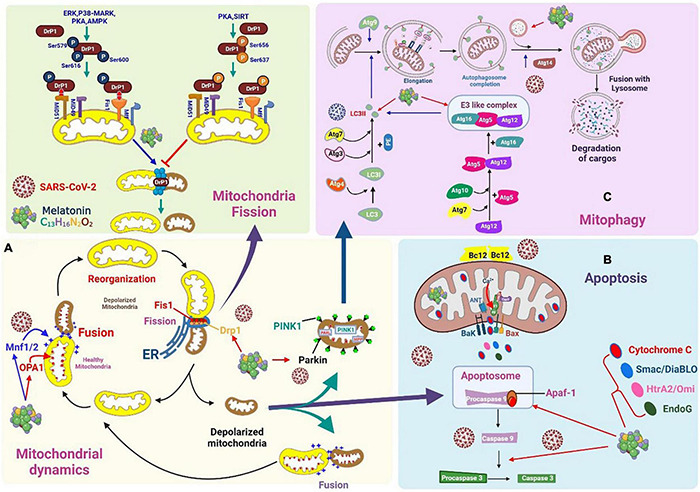
Severe acute respiratory syndrome coronavirus 2 affects mitochondrial dynamics, mitophagy, and apoptosis. **(A)** Mitochondrial fusion is mediated by mitochondrial dynamin-related guanosine triphosphatases, which include mitochondrial fusion proteins 1 and 2. Melatonin downregulates Drp1 expression and suppresses mitochondrial fission. **(B)** Viral protein and open reading frame may induce mitochondria apoptosis. Melatonin may modulate processes associated with apoptosis, which reduces viral spread. **(C)** Coronaviruses can alter mitochondrial dynamics and the status of mitochondrial autophagy to promote interference with the normal antiviral signaling pathway of cells and maintain infection. Melatonin may promote mitophagy via LC3II, autophagosome-lysosome fusion, and E3-like complex.

Mitochondrial dynamics involve repeated fusion and fission cycles within the mitochondrial system. Studies on the monitoring of individual mitochondria during fusion and fission revealed that both events are matched, and fusion triggers fission. The balance between these two opposing processes regulates the shape of the mitochondria and affects mitochondrial quality and homeostasis (117). Previous studies have shown that fusion events coupled to fission event can accelerate the removal of damaged mitochondrial components by autophagy. The fusion process involves mitochondrial membrane components, matrix metabolites, and complete copies of mtDNA that can be rapidly exchanged and equilibrated between adjacent mitochondria. Fusion also repairs and reconstitutes damaged mitochondrial functions. The frequency and selectivity of mitochondrial fusion are essential for maintaining quality (118). In mammals, mitochondrial fusion is mediated by mitochondrial dynamin-related guanosine triphosphatases (GTPases), which include mitochondrial fusion proteins 1 and 2 (Mfn1 and Mfn2, respectively), responsible for fusion of the MOM, and optic atrophy 1 (OPA1), responsible for the fusion of the MIM. In mammalian cells, Mfn2, located both on the MOM and ER surface, has been proposed as a physical bridge between the two organelles. Mfn2 prevents excessive and potentially toxic proximity between both organelles (119) (Figure 5A). OPA1 is located on the MIM, facing the intermembrane space and controlling the fusion and remodeling of the MIM. OPA1 is processed by proteases dependent on ATP levels and Δψm, suggesting that the mitochondrial energy status is an important regulator of OPA1 processing. The cleavage of OPA1 induces mitochondrial dysfunction (120). The mitochondria are rich in the deacetylase SIRT3, which can directly deacetylate OPA1 and activate the protein by increasing its GTPase activity, thereby regulating mitochondrial dynamics under stress (121). Mfn2 in the MOM may mediate the recruitment of parkin to damaged mitochondria. Parkin binds Mfn2 in a phosphatase and tensin homolog-induced kinase 1 (PINK1)-dependent manner. PINK1 (a mitochondrial serine/threonine protein kinase) phosphorylates Mfn2 and subsequently favors parkin-mediated ubiquitination. Experiments have confirmed that the ablation of Mfn2 in mouse cardiomyocytes significantly prevented mitochondrial depolarization-induced parkin translocation and effectively inhibited mitochondrial autophagy (122). Mitochondrial fusion proteins can also be ubiquitylated with multiple E3 ligases and degraded by proteases, which inhibits fusion (123). Mitochondrial depolarization enhances the expression of protease OMA1, which leads to the cleavage and decay of Long-OPA1. This results in a reduction in mitochondrial fusion and the autophagic degradation of mitochondrial organelles (124).

MPTPs are involved in an inducible activity that regulates solute exchange between the mitochondrial matrix and the surrounding cytoplasm. However, this exchange can cause a sharp decline in Δψm, which eventually leads to cell death, whereby the organ is severely swollen, ruptured, and disabled (Figure 5B). The MPTP primarily consists of BAX/BAK on the MOM for inducing membrane permeability. The F1FO ATP synthase on the MIM is regulated by CypD in the matrix and can be used in the response to stimuli for initiating the opening and outflow of Ca^2+^, loss of Δψm, and the swelling and rupture of the mitochondria (125).

Mitochondrial fission is initiated by the recruitment of dynamin-related protein 1 (Drp1) and its anchor points on the MOM. Drp1 is post-translationally modified by phosphorylation, which affects its localization to the cytoplasm or MOM. Extracellular signal-regulated kinases, p38-MAPK, protein kinase A, AMPK, and SIRT are capable of phosphorylating Drp1. The phosphorylation of Drp1 at Ser637 and Ser656 significantly inhibits mitochondrial fission, whereas phosphorylation at Ser616, Ser579, and Ser600 enhances mitochondrial fission (126). Fusion is mediated by mitogens (Mfn1 and Mfn2) on the MOM, and OPA1 plays a major role in MIM. Fission begins when the ER is recruited to the mitochondrial contracting sites, where it is tagged with mtDNA. Next, a variety of MOM-binding proteins (FIS1, MFF, MiD49, and MiD51) recruit Drp1 to the surface of the mitochondria, aiding the contraction response mediated by the ER (127).

SARS-CoV-2 can efficiently inhibit mitochondrial fusion by targeting mitochondrial deubiquitination enzymes to inhibit the deubiquitination of Mfn1 and Mfn2 (128). After SARS-CoV-2 enters the infected cell, viral RNA and proteins are found in the mitochondria. Following infection, non-coding RNA may also regulate mitochondrial dynamic host proteins, such as ubiquitin carboxy-terminal hydrolase 30 (USP30). SARS-CoV-2 may divert the host mitochondria by regulating mitochondrial dynamics, mitochondrial physiological functions, and the release of mtDNA to help suppress the host’s immune response. This diversion is crucial for SARS-CoV-2 infection (128). Drp1 is a key contributor to mitochondrial fission, since its phosphorylation regulates mitochondrial fission (129). Altered mitochondrial autophagy mediated by PINK1/parkin is the molecular basis for mitochondrial anomalies in Parkinson’s disease. Recent studies have shown that PINK1 directly phosphorylates Drp1 at S616. Mitochondrial fission mediated by PINK1 is related to the phosphorylation of Drp1S616 (130). RNA viruses support the proteasomal degradation of Drp1, which contributes to the induction of optimal mitophagy (70). Some coronaviruses augment the ratio of LC3-II to LC3-I, causing autophagosomes to accumulate in infected cells (131). SARS-CoV-2 can target the USP30 protein and inhibit PINK1-mediated mitophagy (132). The different effects of various coronaviruses on host cell autophagy can be attributed to the fact that autophagy inhibits the replication of certain viruses but is beneficial to the replication of other viruses (133).

In other words, fusion-based functional complementarity and mitochondria-derived vesicles can serve as the first line of protection against mitochondrial injury. As the degree of damage to the mitochondria increases, the damaged compartment is separated from the mitochondrial system by fission, followed by mitochondrial autophagy. Depending on the type of stressor, there are many molecular mechanisms responsible for mitochondrial autophagy (127). For example, PINK1/parkin contributes to mitochondrial autophagy when the mitochondria are depolarized. Moreover, when mitochondrial autophagy is induced during hypoxia or erythropoiesis, the connection between damaged mitochondria and new autophagosomes is formed by mitochondrial receptors (for example, NIX, BNIP3, FUNDC1, and BCL2L) interacting with LC3.

### Mitophagy

In the process of mitochondrial depolarization, PINK1 and parkin physically participate and collaborate to identify damaged mitochondria for degradation (134). The completion of specific mitochondrial autophagy (mitophagy) involves several consecutive steps, which are closely regulated by more than 35 genes linked to autophagy (ATGs) and their corresponding proteins (Figure 5C). The first step is the induction of phagophore nucleation, which is affected by a complex of ULK1 (a mammalian homolog of Atg1), Atg13, Atg10, RB1CC1/FIP200, Vps34 (a type III PI 3-kinase), and p150. During the development of mitophagy, the ULK1 complex participates in the initiation of autophagy and the activation of class III PI 3 complexes through the phosphorylation of Beclin-1, which then induces phagophore nucleation (135). The second step is the formation of phagophores and autophagosomes, which is primarily regulated by the Atg9 protein. Thereafter, mitophagy completes the merging of autophagosomes and lysosomes. ATG14 binds directly to the STX17-SNAP29 binary complex on autophagosomes and favors the fusion of autophagosomes and lysosomes by STX17-SNAP29-VAMP8 (136). The final step is the degradation of the cargo trapped in the autophagosome by lysosomal hydrolase. To cope with various conditions of stress, the degraded molecules are transported into the cytoplasm, recycled, and reused to synthesize basic cellular components.

Autophagosome formation is negatively controlled by mTORC1. The inhibition of mTORC1 may lead to the activation of Vps34, which is essential for inducing phagophore nucleation. The translocation of damaged mitochondria to phagophores occurs through the MOM covering the protein, which acts as an LC3-II receptor (137) (Figure 5C). PINK1 and parkin promote the mitochondrial autophagy-mediated degradation of damaged mitochondria (138).

### Effects of SARS-CoV-2 on Mitochondrial Dynamics and Mitophagy

Coronaviruses can alter mitochondrial dynamics and the status of mitochondrial autophagy to promote interference with the normal antiviral signaling pathway of cells and maintain infection. The SARS-CoV-2 ORF3a protein targets USP30, a mitochondrial deubiquitinase that normally binds to the MOM. Because USP30 can counter the effects of parkin, it is a key inhibitor of mitochondrial autophagy. It hydrolyzes the attached ubiquitin and, through parkin, acts on target proteins such as mitochondrial Rho GTPase 1 and Tom20, thereby inhibiting the ability of parkin to drive mitochondrial autophagy (139–142). Additionally, USP30 promotes mitochondrial fusion by deubiquitinating Mfn1 and Mfn2. The expression of mitochondrial ribosomes, respiratory complex I, and mitochondrial fission-promoting genes (such as SOCS6 and MTFP1 genes) is reduced considerably in cells infected with SARS-CoV-2. However, reducing the expression of SOCS6 and MTFP1 limits mitochondrial fission and leads to excessive mitochondrial fusion. This frequently occurs in cells infected with SARS-CoV-2 (143).

Several studies have shown the basic functions of autophagy during a viral infection. Autophagy can induce innate immune responses to inhibit the proliferation of viruses; in contrast, viruses evolve various strategies to defend and escape the destructive effects of autophagy and even use it to promote their proliferation. SARS-CoV-2 rearrangement involves the formation of ER-derived double-membrane vesicles (DMVs) as sites for RNA replication. Phosphatidylinositide 3-kinase, a cytokine with a biosensor, is activated in virus-infected cells to produce phosphatidylinositol 3-phosphate (PI3P), which is closely related to viral replication and the formation of viral DMV. The PI3P-binding protein DFCP1 is recruited to omegasomes during the early stages of autophagosome formation and participates in virus replication and DMV formation. Thus, SARS-CoV-2 uses components of the autophagy machinery to create replicating organelles (144). Similar to porcine epidemic diarrhea virus (PEDV), it can induce an ER stress response, and the activation of NF-κB signaling also contributes to PEDV replication (145, 146). In a study of porcine intestinal epithelial cells, rapamycin-induced autophagy effectively limited the infectivity of PEDV. Therefore, inducing complete autophagy may be an effective strategy for preventing viral infections (131). However, experimental infection with transmissible gastroenteritis coronavirus (TGEV) resulted in an increase in the ratio of LC3-II to LC3-I, leading to the accumulation of autophagosomes in these cells. These results indicate that TGEV infection activates autophagy, and autophagy subsequently inhibits the replication of TGEV (133). Since pharmacological suppression of autophagy can lead to increased replication of TGEV, autophagy induction can be an efficient method to prevent TGEV from causing disease in infected cells. In addition, in TGEV-infected cells, the decrease in total mitochondrial mass, upregulation of mitochondrial p62/SQSTM1 levels, increase in the LC3-II/LC3-I ratio, and expression of Beclin 1 implies that TGEV may induce mitochondrial autophagy to attenuate oxidative stress and cell apoptosis, which will further promote cell survival and possible viral infection. In simple terms, mitochondrial autophagy in TGEV infection can counteract oxidative stress and apoptosis and help the virus to replicate (147). Since autophagy is critical for SARS-COV2 mediated COVID-19, we can design drugs that target autophagy to inhibit and treat viral infections (148).

SARS-CoV-2 is also detrimental to other special characteristics of autophagy in infected cells (Figure 5C). Its ORF9b protein localizes in the mitochondria, which accelerates the degradation of Drp1, thereby prolonging the lifespan of the mitochondria. ORF9b may also target and degrade important mitochondria-binding molecules, such as MAVS, TRAF3, and TRAF6, which severely restricts the antiviral IFN response in the host cell (90, 149).

The host USP30 protein is pivotal for mitochondrial homeostasis and collaborates with parkin-mediated mitochondrial autophagy. The ORF3a protein of SARS-CoV-2 can interact with USP30 to alter mitochondrial ubiquitination, causing a mitochondrial collapse in infected cells and premature death (90). Previous studies have reported that infection with SARS-CoV-2 upregulates the expression of PINK1, which contributes to the development of mitophagy (150). However, the expression of the E3 ubiquitin ligase Skp2 is significantly reduced in cells infected with SARS-CoV-2, which prevents the degradation of Beclin 1, which may induce significant autophagic flow (128, 151). The similarity in the amino acid sequence of ORF9b in SARS-CoV-2 and SARS-CoV shows that SARS-CoV-2 ORF9b may inhibit mitochondrial breakage and induce autophagosome formation to promote cell survival and increase virus infection and replication. However, co-expression network analysis shows that SARS-CoV-2 reduces the autophagic flux by upregulating glycogen synthase kinase 3 beta or downregulating autophagy genes and lysosomal acidification genes (128, 143).

### Effect of Melatonin on Mitochondrial Dynamics and Mitophagy

Melatonin downregulates Drp1 expression and suppresses mitochondrial fission (Figure 5A). This effect can be mediated *via* the activation of the SIRT1–PGC-1α pathway because the combination of PGC-1α and its promoter decreases the expression of Drp1. During viral infection, the activation of SIRT1 increases Δψm and reduces ROS production. Since melatonin is absorbed and synthesized in the mitochondria, this is the best method to suppress ROS production (152). Melatonin improves mitochondrial function and biogenesis by inducing the expression of SIRT1 (153) and can even inhibit neural damage caused by prions by regulating mitochondrial function and dynamics. Melatonin corrects the imbalance in mitochondrial dynamics by enhancing OPA1 and reducing Drp1 expression (154). Further, it decreases the translocation of Drp1 in mitochondria, thus attenuating the co-localization of Drp1 and Tom20 proteins (155). Melatonin also inhibits mitochondria-mediated cell death by preventing the opening of MPTP and activating PINK1/parkin, thereby inhibiting mitochondrial Drp1-mediated fission.

AMPK directly promotes autophagy by enhancing the phosphorylation of autophagy-related proteins (mTORC1, ULK1, and PIK3C3/VPS34 complexes). Further, it can indirectly promote autophagy by regulating transcription factors such as BRD4, FOXO3, and TFEB. AMPK causes the fragmentation of damaged mitochondria and favors the translocation of autophagic machines to damaged mitochondria to achieve effective mitophagy (156). A porcine intestinal epithelial cell study (IPC-J2) has shown that AMPK-mediated mitophagy is needed to attenuate the lesions of the intestinal epithelial barrier caused by oxidative stress and dysfunction of mitochondrial energy metabolism (146). In addition, AMPKα can inhibit mitochondrial fission by inhibiting Drp1 (157). The inhibitory effect on Drp1 activation and mitochondrial translocation of melatonin may be owing to the antioxidant effect of melatonin and its prevention of cytosolic calcium overload. Melatonin can also activate AMPKα, which inhibits the opening of the MPTP (158). Moreover, under oxidative stress, melatonin restores the reduced expression of Mfn2 and OPA1 by activating the AMPK/PGC-1α pathway to achieve efficient mitochondrial fusion (110) (Figure 5A).

## Apoptosis and Mitochondrial Quality Monitoring

**FIGURE 6:**
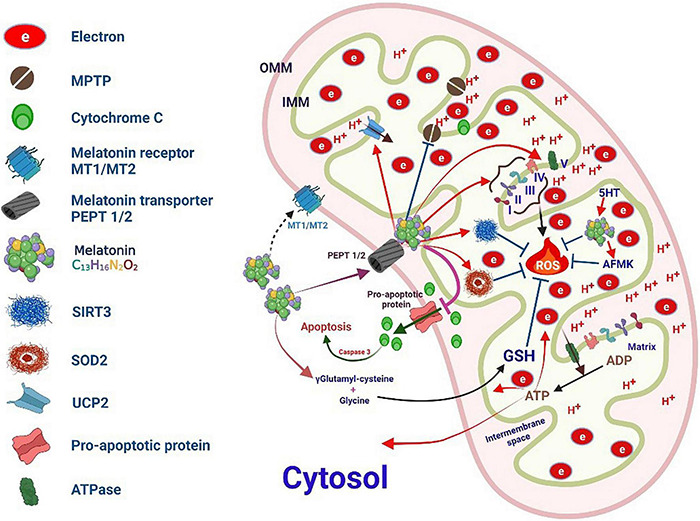
Melatonin targets mitochondrial oxidative stress.

The fusion of healthy and defective mitochondria can help repair dysfunctional mitochondria. However, when mitochondrial damage is severe, dysregulated mitochondria can be separated by fission, and these separated organelles are eventually eliminated by mitophagy. Defective mitochondria may rupture if quality control is compromised or mitochondrial damage exceeds the reparative capability of fission/fusion and mitophagy routes (159). When the mitochondria are damaged, apoptotic factors are released from the damaged mitochondria into the cytoplasm, which triggers the death of apoptotic cells by inducing caspase activation, chromosomal condensation, and rupture. These apoptotic factors are released when members of the pro-apoptotic Bcl-2 family destroy the integrity of the MOM. When these pro-apoptotic proteins, including Bax and Bak, are activated, they form the MPTP, which leads to the penetration of the MOM (160). This process releases various apoptotic proteins and cytochrome c from the MIM space into the cytoplasm (159). In the cytoplasm, cytochrome c interacts with the activator of apoptotic protease 1 and procaspase-9 to produce apoptosome complexes. Apoptotic bodies are subjected to the autocatalytic activation of caspase-9, followed by activation of caspase-3. Once activated, caspase-3 cleaves vital cellular proteins and subsequently induces cell death (161). Caspase-3 is a critical intracellular proteolytic enzyme that cleaves several key substrates during apoptosis, causing DNA fragmentation and nucleoprotein degradation and promoting apoptotic body formation (Figure 6) (162). In addition to caspases, cell death factors released by the mitochondria can activate caspase-independent cell apoptosis. Furthermore, the apoptosis-inducing factor, a mitochondrial flavoprotein, is translocated into the nucleus to bind to chromosomal DNA and cause chromatin condensation, which causes DNA fragmentation (163). In addition, Bax/Bak promotes Drp1 phosphorylation, inducing a stable membrane-associated form of Drp1 during apoptosis.

### Effects of SARS-CoV-2 Infection on Mitochondrial Apoptosis

Intracellular BCL-2-related proteins (BCL-2s) promote the mitochondrial release of pro-apoptotic signaling molecules to regulate apoptosis. These factors activate cysteine proteases of the caspase family, which help propagate the apoptotic cell death signal (164). Correspondingly, the inhibition of the apoptosis of infected cells can increase the risk of viral infection. For example, during mouse hepatitis viral infection, the expression of the pro-apoptotic gene BNIP3 is downregulated in cultured astrocytes (165).

The caspase recruitment domain (CARD) is involved in the cascade of apoptotic and inflammatory signals. Upon activation, Nod-like receptors assemble into multi-protein complexes, such as NODosomes and inflammasomes (166). When activated by RNA viruses, MAVS initiates type 1 IFN signaling by activating the nuclear translocation of NF-κB and IRF3. MAVS, a mitochondrial antiviral-signaling protein, possesses an N-terminal CARD domain, which indicates that the mitochondrial localization of MAVS is related to the induction of apoptosis (167). Therefore, the inhibition of MAVS function is an effective strategy for some viruses to inhibit the death of host cells (166). The NSP15 protein of SARS-CoV assists in the replication of SARS-CoV, and can completely inhibit MAVS-induced apoptosis (166). NSP15 of murine coronavirus inhibits RIG-I and MDA5 sensors for the efficient monitoring of viral dsRNA, thereby delaying IFN activation and inhibiting apoptosis in infected macrophages (168).

Many viral gene products can effectively trigger apoptosis by interfering with cellular signaling cascades. Previous studies have shown various pro-apoptotic proteins in the SARS-CoV genome, including ORF3a, ORF3b, ORF4, ORF6, ORF7a, and ORF7b, which can induce apoptosis in specific infected cells (169, 170). In cells infected with SARS-CoV, the ORF3a protein is found in the ER, which can induce chromatin condensation as well as DNA fragmentation, leading to host cell apoptosis (171), which favors mitochondrial apoptosis by activating p38 MAPK signaling (172). SARS-CoV-2 ORF3a can effectively induce host cell apoptosis by promoting caspase-3 activation, Bid truncation, caspase-9 lysis, and cytochrome c expression (173). SARS-CoV 3b protein (ORF4) is present both in the mitochondria and nucleus during viral infection, and its overexpression through transfection can induce cell cycle arrest in the G0/G1 phase (170). Studies have confirmed that transient transfection of green fluorescent protein (GFP)–ORF6 in different cells induces host cell apoptosis by promoting the activation of caspase-3 and c-Jun N-terminal kinase (JNK)/MAPK pathways, which are elicited by the exacerbation of ER stress. In addition, the level of the ER chaperone protein GRP94 was significantly upregulated (169). In the ER, ORF7a can act on the anti-apoptotic protein Bcl-XL, causing the isolation of Bcl-XL in the ER-Golgi apparatus and subsequently promoting apoptosis (174), associated with the activation of p38 MAPK (175). The ORF7a protein may also interact with human Ap4A hydrolase, triggering the activation of the cascade mechanism that causes cell cycle arrest and apoptosis (176). The SARS-CoV M protein induces apoptosis by lowering Akt pro-survival signaling and inducing the release of mitochondrial cytochrome c (177). ORF8a is primarily located in the mitochondria and not only promotes virus replication, but also accelerates mitochondrial hyperpolarization, ROS production, and subsequent caspase 3-dependent apoptosis (Figure 5B) (178).

SARS-CoV-2 also induces apoptosis in various lymphocytes, which is related to the uncontrolled production of inflammatory cytokines, leading to excessive inflammation and eventually causing damage to visceral organs (179, 180). In patients with acute COVID-19, non-clonal T cell populations are characterized by the upregulated expression of voltage-dependent anion channels and mitochondrial membrane (mtM) proteins. These mtM proteins are involved in enhanced permeabilization of the MOM, which induces infected cell death. The increase in mtM protein and anion channel expression is also related to mitochondrial dysfunction, which leads to the release of cytochrome c and caspase activation. Finally, caspase-induced T cell apoptosis can lead to decreased lymphocyte count, which is observed in patients with COVID-19 (181, 182).

### Melatonin and Mitochondrial Apoptosis

Melatonin plays an entirely different role in healthy cells than in tumor cells. Melatonin interfaces with cancer cells to promote apoptosis (183) while preventing apoptosis in healthy cells (184). Melatonin has the ability to improve immune surveillance, recover free radicals, which significantly reduces associated molecular harm, and modulate processes associated with apoptosis, which reduces viral spread (185) (Figure 5B).

The anti-apoptotic properties of melatonin are primarily related to the inhibition of MPTP opening and Ca^2+^ overload induced by oxidative stress. The overloading of Ca^2+^ subsequently inhibits the depolarization of the mitochondrial membrane, facilitates the opening of the MPTP, and induces the release of Ca^2+^ (186) (Figure 6). In addition, melatonin can stabilize the level of Ca^2+^ buffer proteins such as paralbumin and hippocampal calcineurin, which prevents a large increase in intracellular Ca^2+^ levels and possibly induces apoptosis (187). Melatonin can also donate or accept electrons from the mitochondrial ETC complex, which enhances the activity of respiratory complexes and helps maintain effective oxidative phosphorylation and ATP synthesis (188). Melatonin influences these processes to improve mitochondrial respiration and energy production, thereby reducing oxygen consumption and superoxide anion production, depolarizing mitochondria, causing MPTP opening, and releasing apoptotic proteins and cytochrome c from the MIM space to the cytoplasm to reduce oxidative damage (189). Melatonin antagonizes injury-induced apoptosis by interacting with MT-1 and MT-2 plasma membrane receptors. In response to apoptotic stimuli, melatonin allows mitochondrial translocation of the pro-apoptotic protein Bax but impairs its activation/dimerization and prevents the formation of MPTP in downstream apoptotic events. Melatonin also induces the strong relocalization of Bcl-2, a major Bax antagonist in mitochondria, thereby antagonizing the intrinsic pathway of apoptosis (190). Further, it can downregulate the phosphorylation of JNK and p-38 MAPK by activating the membrane melatonin receptors MT1 and MT2 to inhibit p53 phosphorylation (191). In addition, melatonin binds to the MT1 receptor located on the MOM and activates the MT1/Gαi signaling in the mitochondrial intermembrane space, effectively inhibiting the activity of adenylate cyclase and Ca^2+^-mediated cytochrome c release and preventing caspase activation and subsequent apoptosis (192).

A previous report showed that melatonin exerts a significant anti-apoptotic effect in cells with certain viral infections, such as the Venezuelan equine encephalitis (VEE) infection (193). Melatonin also inhibits hepatocyte apoptosis induced by rabbit hemorrhagic disease virus, primarily owing to the upregulation of Bcl-2 and Bcl-XL expression, downregulation of Bax expression, reduction of cytochrome c release, and restriction of caspase-9 activation (194). Moreover, melatonin restores mitochondrial function in coxsackievirus B3 (CVB3)-infected cardiomyocytes. Further, melatonin significantly improved CVB3-induced myocarditis, inflammatory cell infiltration, necrosis, and edema, and decreased the expression of autophagy-related proteins, thus inhibiting apoptosis (195).

## The Effect of Melatonin on the Redox State of the Mitochondria

Because of its lipophilic and hydrophilic properties, melatonin can easily diffuse into cells and intracellular organelles and cross the blood-brain barrier. The pyrrole ring in melatonin makes it highly suitable for capturing O_2_^•–^ and ^•^OH free radicals. Previous studies have demonstrated that melatonin is five times more effective at scavenging free radicals than GSH, an endogenous free radical scavenger, and 15 times more effective than mannitol, an exogenous free radical scavenger. Its antioxidant effect is primarily mediated by the capture of O_2_^•–^ radicals by indole cation radicals formed by the reaction of melatonin and ^•^OH radicals (14, 196). In particular, melatonin is abundant in subcellular compartments, such as the nucleus and mitochondria (197, 198).

Further, mitochondrial dysfunction is extremely critical for the processes of cellular self-destruction, including apoptosis, autophagy, and necrosis, during various diseases (199). mtDNA is more fragile than nuclear DNA because it lacks histones. Therefore, mtDNA damage in the elderly is ten times more severe than nuclear DNA damage (199). The mitochondria are well-known as the prime source of ROS formation but are also the prime targets of attack by ROS (2) (Figure 6).

A previous report showed that melatonin treatment prevents the age-dependent increase in mitochondrial oxidative stress (200). Melatonin is also essential for mitochondrial homeostasis as it optimizes the transfer of electrons to the ETC in MIM (201, 202). Another study showed that melatonin treatment restored mitochondrial ATP production in cardiomyocytes. Further, long-term melatonin therapy protects against mitochondrial dysfunction and oxidative stress without any side effects (203).

Under normal circumstances, organisms possess powerful defense mechanisms against oxidative stress, one of which involves the use of antioxidant enzymes. However, the potency of antioxidant enzymes gradually declines with age (204, 205). An age-dependent increase in oxidative stress reduces the mitochondrial GSH pool in the brain by 50% and simultaneously increases oxidative GSH levels (206, 207). A study in rat lymphocytes and brain showed that reductions in components related to free radical scavenging, such as catalase and SOD activities and GSH levels, were associated with aging (208). Although the mitochondrial antioxidant defense mechanisms are dependent on GSH, the mitochondria cannot synthesize GSH (209). Instead, GSH is imported into the mitochondria from the cytosol through a multicomponent transport system (210). Melatonin increases GSH-Px, GSR, SOD1, and CAT gene expression and enzymatic activity at pharmacological and physiological doses (211). The antioxidant enzymes GSH-Px and SOD are critical for protecting the body from age-associated oxidative stress. GSH-Px activity is known to increase with age, whereas melatonin therapy was shown to decrease GSH-Px activity in elderly rats (56). However, melatonin treatment augmented the mitochondrial SOD levels in older rats and prevented the reduction of the SOD/GSH-Px and GR/GSH-Px ratios (212). Melatonin treatment in rats with renal ischemia/reperfusion lesions increases SOD activity and GSH levels and decreases nitrogen oxide levels (2, 213).

The mitochondrial respiratory chain primarily uses oxygen as raw material and produces energy as ATP. Free radicals and ROS are formed when electrons transferred between consecutive complexes chemically reduce adjacent oxygen molecules. The complexes involved in oxidative phosphorylation in the ETC (CI–CV) are then formed, which produces the energy necessary for the cell in the form of ATP. Melatonin enters the mitochondria *via* the peptide transporter (PepT 1/2) in a reverse gradient. Further, we found that the melatonin concentration in mitochondria is considerably higher than that in other subcellular compartments (152).

Melatonin plays a multifaceted role in the mitochondria. In particular, it strengthens the tolerance of key mitochondrial molecules to oxidative damage and preserves the function of healthy organelles. ROS generated *via* electron leakage from the ETC can be directly eliminated by melatonin and its metabolite [N1-acetyl-N2-formyl-5-methoxykynurenure (AFMK)]. Indeed, ROS can also be metabolized by mitochondrial superoxide dismutase (SOD2) and subsequently scavenged by GSH and SIRT3. In addition, melatonin can effectively modulate uncoupling protein 2, maintain optimal MIM potential, and effectively limit the opening of MPTP. In addition to its rapid entry into the mitochondria, melatonin can also be synthesized in the mitochondria, where it can be metabolized to AFMK; therefore, it has optimal properties for eliminating toxic oxidative substances (152).

## Melatonin Supplementation for COVID-19

A retrospective review of intubated patients with COVID-19 revealed that melatonin reduced the risk of death considerably, indicating a significant anti-inflammatory effect. Melatonin can upregulate the expression of SIRT1, a deacetylase known to effectively inhibit the activity of pro-inflammatory NF-κB, and can also upregulate Nrf2, which in turn increases antioxidant protein transcription (214, 215). Recent epidemiological studies have shown that melatonin supplementation can reduce the risk of COVID-19 infection detected serologically by 28%, a significant reduction; among Black Americans, this risk is reduced by 52% (216). The basis for this risk reduction may be related to the enhancement of SIRT1 activity by melatonin and the subsequent transcriptional upregulation of ACE2 expression (217).

SIRT1 induced by melatonin can enhance virus-mediated MAVS activation (218). Through mitochondrial membrane receptors, melatonin induces the nuclear translocation of the transcription factor retinoid-related orphan receptor alpha (RORα). RORα boosts the transcription of the gene encoding the clock transcription factor brain and muscle aryl hydrocarbon receptor nuclear transporter-like 1 (Bmal1). Bmal1 transcription significantly upregulates the expression of SIRT1 and Nrf2 (214) (Figure 4). Nrf2 plays a pivotal role in the innate immune system by inhibiting ROS and directly inhibiting the pro-inflammatory cytokines IL-1β and IL-6 to limit the development of inflammation. In macrophages, Bmal1 regulates IL-1β *via* Nrf2 (219). The MAVS protein is a key mediator in the dsRNA sensing pathway, which leads to the activation of IRF3 and the induction of type 1 IFNs (220). TRIM31 is an E3 ubiquitin ligase of the TRIM protein family and is mainly used as a regulator of MAVS aggregation. TRIM31 is recruited to the mitochondria following viral infection and specifically regulates antiviral signal transduction mediated by RLR PRRs. TRIM31 interacts with MAVS and catalyzes its Lys63 (K63)-linked polyubiquitination at Lys10, Lys311, and Lys461. This modification leads to the formation of MAVS prion-like aggregates following viral infection, promotes the activation and phosphorylation of IRF3, and induces type 1 IFN (221) (Figure 4). However, ovarian tumor ubiquitinating enzyme 3 (OTUD3) suppresses this activation by deubiquitinating MAVS (222). In response to a viral infection, acetylated Lys129 is deacylated and eliminated by SIRT1, which rapidly inactivates OTUD3, inducing innate antiviral immunity by upregulating the viral activation of MAVS and inducing type 1 IFN (222).

The effect of SIRT1 on IFN-induced antiviral immunity is complex, as SIRT1 may inhibit NF-κB transcriptional activity. NF-κB also works downstream of MAVS to promote type 1 IFN induction (223). The cell’s response to RNA viruses often activates IRF3, NF-κB, ATF2, and c-Jun, which can bind to the promoter of the IFN-β gene IFNB1 and promote its transcription (219).

Nrf2 is a sensor of oxidative stress that maintains a normal physiological state by inducing redox balance. Melatonin mediates various beneficial biological and therapeutic effects, such as antioxidant, anti-inflammatory, anti-tumor, anti-diabetic, and cardioprotective effects. The Nrf2 signaling pathway explains some of the therapeutic and biological effects of melatonin (224).

**FIGURE 7:**
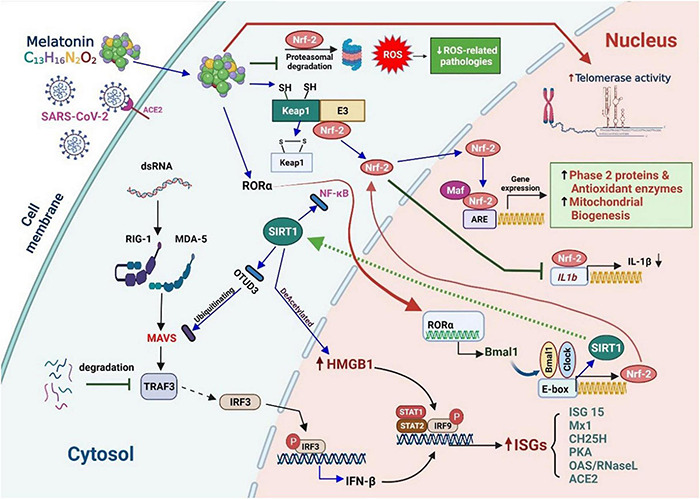
Melatonin supplementation reduces the risk of coronavirus disease (COVID-19) by rescuing the repression IFN/ISG production. Melatonin supplementation restores the suppressed production of IFN/ISG, increases telomerase activity, upregulates SIRT1 expression, enhances virus-mediated MAVS activation, promotes nuclear translocation of the transcription factor RORα, enhances Bmal1 expression, and further upregulates SIRT1 and Nrf2 expression to reduce the risk of COVID-19. Cellular responses to RNA viruses tend to activate IRF3, NF-κB, ATF2, and c-Jun, which may bind to the promoter of the IFNB1 gene and promote its transcription. Overall, melatonin can help suppress processes that promote inflammation, such as NO production, cyclooxygenase 2 activity, NLRP3 inflammasome formation, and cytokine release, in elderly patients with COVID-19. It also activates processes in the anti-inflammatory network, including SIRT1 activation, Nrf2 upregulation, NF-κB downregulation, and release of the anti-inflammatory cytokines IL-4 and IL-10.

In most vertebrates, melatonin synthesis gradually decreases with age. This decrease may be attributed to a decrease in β-adrenergic receptors in the pineal gland and in the expression of aralkylamine N-acetyltransferase (AANAT) (225). Older cells are prone to produce more reactive nitrogen species (RNS) and ROS, but the antioxidant effect of endogenous melatonin counteracts the damage caused by RNS and ROS in senescent cells (3). Previous studies have shown that melatonin can retard the aging of the gastric mucosa by stimulating telomerase activity and effectively inhibiting lipid peroxidation and cell proliferation (226). Melatonin can also reduce oxidative damage induced by angiotensin by inhibiting the production of ROS and RNS, inflammatory cytokines, and advanced glycation end products and preventing telomere shortening (26) (Figure 7).

Respiratory epithelial cells, macrophages, monocytes, and dendritic cells can produce IFN. RIG-1/MDA-5 can recognize viral dsRNA in the infected cytoplasm and help TRAF-3 activate IRF3. Aging is related to the degradation of TRAF-3 and reduction in the phosphorylation of IRF3. IRF3 acts as an intermediary for the transcription of IFN-I and IRF8. IRF8 enhances and amplifies IFN-I expression. The deubiquitination of MAVS by OTUD3 can inhibit the activation of IRF3, thus blocking the innate antiviral immune response (222). SIRT1 can remove the OTUD3 acetyl group, switch off OTUD3 activity, and thus upregulate the viral activation of MAVS and type 1 IFN (222). In response to inflammatory signals or certain viral infections, the DAMP protein high-mobility group box-1 (HMGB1) is over-acetylated, causing it to be exported from the nucleus and released from the cell (227). After reversing the acetylation, SIRT1 tends to confine HMGB1 to the nucleus and promote the transcription of antiviral genes stimulated by type 1 IFNs in the nucleus (228). Oxidative stress causes Keap1 to release Nrf2, following which Nrf2 is phosphorylated and translocated into the nucleus. Melatonin may inhibit Nrf2 ubiquitination, thereby suppressing its proteasomal degradation. In the nucleus, Nrf2 binds to Maf, which binds to the antioxidant response element (ARE). This leads to the transcription of many phase 2 enzymes, which convert ROS and RNS into inactive products, thus reducing oxidative stress (219). Phase 2 enzymes traditionally refer to enzymes that catalyze the conjugation reaction, such as GSH S-transferase. However, the scope of the term has gradually expanded to include several enzymes that catalyze the phase 1 reaction, such as heme oxygenase-1 and NAD(P)H:quinone oxidoreductase type 1.

In short, melatonin can regulate the induction of type 1 IFN and ISGs by inhibiting OTUD3 and promoting the nuclear retention of HMGB1. It also inhibits NF-κB activity and enhances Nrf2 signaling. Furthermore, melatonin improves Keap/Nrf2/ARE signaling and can inhibit the metabolic pathways of ubiquitin and proteasomes (111, 229).

## Melatonin Reverses Biological Aging

### Melatonin and the Mitochondria

The mitochondria play a key role in calcium homeostasis, apoptosis, and the regulation of multiple physiological and pathological processes (230). They are responsible for energy production (ATP) *via* glycolysis in the cytosol and oxidative phosphorylation in the MIM. Glycolysis produces pyruvate, which is actively transported to the mitochondrial matrix, where it is metabolized to acetyl-CoA (231). The mitochondrial ETC uses a series of electron transfer reactions to generate cellular ATP by oxidative phosphorylation. One consequence of electron transfer is the generation of ROS, which contribute to both homeostatic signaling as well as oxidative stress during pathology (232). Acetyl CoA is also a significant co-factor of AANAT, which converts serotonin to N-acetylserotonin, a precursor of MT (233). The presence of nitric oxide synthase (mtNOS) in the mitochondria indicates the production of nitric oxide (NO) and peroxynitrite (ONOO^–^), which are generated in these organelles during inflammation (234, 235). Additionally, the mitochondria produce precursors of DNA/RNA, proteins, and lipids (236), and participate in the maintenance of cellular homeostasis by Ca^2^**^+^** (237). Conversely, the mitochondria regulate apoptosis by releasing apoptotic factors (cytochrome C) and activating caspases (230, 238).

Melatonin synthesized in the mitochondria provides protection against oxidative stress and the reprogramming of altered intracellular metabolism to levels higher than those in other subcellular organelles and in the absence of circadian rhythms (197, 239). Mitochondria synthesize melatonin *de novo* and release it, which controls the release of cytochrome C in an autocrine-regulated manner through the MT1 receptor on the membrane (240). Melatonin is present in mitochondrial membranes and passes into the mitochondria through the oligopeptide transporters PEPT1 and PEPT2 located on the membrane (241). The Mitochondrial Free Radical Theory of Aging proposes that mitochondrial free radicals, produced as by-products during normal metabolism, cause oxidative damage (242). The effects of aging include mitROS production, lipid unsaturation, autophagy, mtDNA repair, and others, such as apoptosis and protease or telomeric shortening, corresponding to the various theories of aging (243). These changes exert severe effects on mitochondrial and cell physiology and are common in mitochondrial diseases (244, 245). High levels of melatonin and its multiple actions as an antioxidant provide strong protection to the organelles exposed to free radicals (152). Within the mitochondria, melatonin acts as a direct scavenger of free radicals and related non-radical products, stimulates antioxidant enzymes, including SOD2, catalase, and glutathione reductase, and simultaneously inhibits pro-oxidative enzymes (13, 246, 247).

Melatonin, *via* its receptor-independent activities, may suppress NO synthesis and lipoxygenase activity, which aids the formation of superoxide anion (248). By controlling lipoxygenase activity, melatonin protects cells from the hydroperoxidation of polyunsaturated fatty acids (249). It also modulates reactions associated with ER stress (250), sirtuin activity (251), mitophagy, and autophagy (252).

Melatonin also possesses a significant ability to enhance the efficiency of the ETC and improve the production of ATP (253). The marked improvement in mitochondrial function in response to melatonin reduces ROS production, thereby preventing a deleterious reduction in the mitochondrial membrane potential (189, 254). Low melatonin levels may lead to serious consequences in terms of increased oxidative stress and reduced ATP production, followed by increased ROS formation (152). Melatonin also confers multiple layers of protection to the mitochondria *via* its metabolites, such as cyclic 3-hydroxymelatonin (C3-OHM) and AFMK; these allow melatonin to extend the metabolic cascades necessary for mitochondrial protection during mROS- and mCa^2+^-mediated MPTP-associated apoptotic stress, and may provide therapeutic benefits in neurodegenerative diseases (255).

### Melatonin Reverses Mitochondrial Biological Aging

Aging and various age-related diseases are associated with multiple factors, including reduced secretion of hormones such as melatonin (256), reduced activities of aging-related proteins, such as SIRT1 (239, 257), deterioration of circadian oscillator system activity (258), multiple alterations in the immune system with frequent shifts toward the proinflammatory arm (259), and several other deviations from regular biological functions of cells. Notably, the changes are largely based on the pleiotropy of melatonin (260) and the circadian system (261).

In general, melatonin is an immune stimulant, and the direction in which changes in balance have turned out to be highly conditional. The effect of melatonin on the expression of SIRT1 also revealed downregulation or upregulation, in which case a marked difference was observed between tumor and non-tumor cells (258). The activities of sirtuin are not primarily determined by their protein levels but by the NAD^+^ concentration, which depends on the activity of nicotinamide phosphoribosyltransferase (NAMPT) (262, 263). SIRT1 activity, but not its level, was found to be reduced because of lowered NAD^+^ levels in the course of aging (264). The high SIRT1 expression levels in several age-related diseases highlight the importance of epigenetics in healthy aging (264). A number of studies have shown that melatonin increases the expression of SIRT1, which could be suppressed by sirtuin inhibitors (265, 266). Pretreatment with melatonin reversed the dexamethasone-induced negative effects on matrix degeneration in chondrocytes. The significant decrease in the NAD^+^/NADH ratio and NADPH concentration in the dexamethasone group was reversed upon pretreatment with melatonin (266).

Overall, melatonin inhibits inflammation-promoting processes during aging, such as NO release; cyclooxygenase 2, NLRP3inflammasome, gasdermin D, TLR4, and mTOR signaling; senescence-associated secretory phenotype induction; cytokine release; and beta-amyloid toxicity. It also activates processes in the anti-inflammatory network, which involve the activation of SIRT1, upregulation of Nrf2, downregulation of NF-κB, and the release of the anti-inflammatory cytokines IL-4 and IL-10. Another key role may be to promote the polarization of macrophages or microglia, which favors the anti-inflammatory phenotype of M2 macrophages.

Clinical network analyses, which compare medications used to treat SARS-CoV-2 in humans, have also predicted that melatonin would be the most effective agent to prevent/treat COVID-19. Importantly, melatonin use is associated with a 52% reduction in the likelihood of a positive laboratory test result for SARS-CoV-2 (216). When patients severely infected with COVID-19 were treated with melatonin, the severity of infection, mortality, and length of hospitalization were reduced. Of note, melatonin has a high safety profile at a broad dose range and is not highly toxic (267). Consequently, we encourage the consideration of the use of melatonin as a countermeasure to SARS-CoV-2 infection. Melatonin plays a crucial role in maintaining normal mitochondrial function and energy production in cells by directly scavenging ROS in the mitochondria, activating antioxidant production, and protecting membrane integrity, especially in elderly patients. Further research into the effects of melatonin on the mitochondrial function in patients with COVID-19 as well as mitochondria-targeted melatonin studies will help provide insights into the mechanisms underlying its protective effects and suitability for use in the treatment of various diseases.

## Potential Adverse Effects of Melatonin in the Elderly

### Dual Immune Effects of Melatonin in Viral Infection

Melatonin is an immunomodulating agent with pro- and anti-inflammatory properties. Its pro-inflammatory properties have been well-documented in several studies of leukocyte-derived cell lines; therefore, melatonin may be detrimental in autoimmune diseases (215), rheumatoid arthritis (268), and multiple sclerosis (269). The immune effects of melatonin are condition-based, although the conditions are not fully understood (270, 271). Melatonin exhibits pro-oxidant, pro-apoptotic, and SIRT1-downregulating properties in cancer cells. However, melatonin is well-known as an antioxidant and anti-apoptotic agent in most non-tumor cells and acts as a SIRT1-upregulating agent under senescent conditions (239, 258). Chronic low-grade inflammation is a hallmark of aging (272). It is generally accepted that melatonin primarily exhibits anti-inflammatory activities (215). As a result, its usage in an aging population is reasonable.

### Beneficial Effects of Melatonin in COVID-19

Recent reports suggest that melatonin has been proposed to overcome sepsis-related hyper-inflammation with extensive oxidative damage and cytokine storm in SARS-CoV-2 infection (273, 274). During SARS-CoV-2 infection, viral ORF3a induces HIF-1α activation, which in turn aggravates viral infection and inflammatory responses (275). Melatonin may attenuate the damage resulting from COVID-19-mediated septicemia by mollifying HIF-1α, suppressing NF-κB, inhibiting the inflammasome, converting pro-inflammatory M1 macrophages to anti-inflammatory M2 macrophages, and reversing Warburg-type metabolism (276–278). After administration as an adjuvant therapy, melatonin was found to attenuate Th1 and Th2 inflammatory cytokines by downregulating the expression of Th1 and Th2 regulatory genes in patients with COVID-19 (279). A correlation between the levels of circulating secreted Group IIA phospholipase-A2 (SPLA2-IIA) and the severity of COVID-19 was recently reported (280). Activated sPLA2-IIA is an inflammatory agent that is particularly damaging to cellular bio-membranes owing to its ability to hydrolyze fatty acids (281). The damaged membrane releases arachidonic acids that are then transformed through cyclooxygenase into different eicosanoids. These eicosanoids stimulate inflammation and oxidative stress in multiple organs (282, 283). Given the known properties of sPLA2-IIA, it may prolong and exacerbate tissue and organ damage in COVID-19. Commonalities between the symptoms of different conditions associated with sPLA2-IIA may explain some of the beneficial effects of melatonin observed in various diseases.

### Adverse Effects of Melatonin

The maximal effective doses of melatonin range from 0.5 to 10.0 mg (284). The European Food Safety Authority (EFSA) recommends maximum doses of 0.3 to 1.0 mg (285). Although pharmacopeias and the EFSA recommend administering melatonin 1–2 h before bedtime, several studies have shown that melatonin is not always effective if administered according to this recommendation (285). It is mainly metabolized in the liver (90%), in a process primarily involving CYP1A2. The most common side effects are headaches, nausea, and dizziness (286). Exogenous melatonin can reduce blood pressure and cause hypothermia in the elderly (286). A large study reported the side effects associated with the use of melatonin, including neurological disorders in 43%, anxiety and depression in 24%, rashes in 19%, and digestive problems in 19% of the study participants (287).

Additionally, drug interactions with melatonin are a source of concern. Cardiovascular diseases are more common in the elderly, and beta-adrenergic receptor blockers are often used for treatment (288). Beta-blockers reduce melatonin production by significantly inhibiting β-1 adrenergic receptors (289). Melatonin intake before bedtime can prevent this common side effect in beta-blocker users (290). Previous studies have reported that melatonin inhibits glucocorticoid synthesis (291). Melatonin exerts inhibitory effects on adrenocorticotropic hormone secretion in the anterior pituitary and on adrenal cortisol production (292). Melatonin also reduces glucocorticoid-induced toxicity by reducing glucocorticoid receptor nuclear translocation (293).

## Conclusion

This review discusses the benefits of melatonin intake in elderly patients with COVID-19. After the viral protein enters the mitochondria, it uses various mitochondrial protein delivery systems and host cell accessory proteins to stabilize itself. The mitochondria may also complete the steps required to replicate the virus through the viral protein translocation machinery, UPS, and mitochondrial electron distribution system, thereby severely aggravating oxidative stress and inflammation. In addition, COVID-19 affects mitochondrial fusion and fission, which enlarges the mitochondria. Damaged mitochondria undergo mitophagy or apoptosis. However, melatonin plays an important role in these processes and can adequately mitigate these conditions.

Melatonin has the potential to reduce inflammation and possibly curb cytokine storms caused by SARS-CoV-2. Melatonin can directly scavenge various toxic oxygen- and nitrogen-based reactants, stimulate antioxidative enzymes, increase the efficiency of the ETC (thereby limiting electron leakage and free radical generation), and promote ATP synthesis (209). Melatonin is also anti-inflammatory and has special medicinal value in conditions associated with severe inflammation, such as sepsis and ischemia/reperfusion, and low-grade inflammation during aging (215).

Further, the effects of melatonin seem to be closely related to the upregulation of SIRT1. Melatonin also interferes with mTOR and Notch signaling and reduces the expression of pro-inflammatory lncRNA-CCL2 (218). Compared with younger adults, the elderly are more likely to contract severe COVID-19, and the degree of inflammation and aberrant immune response is closely related to the mitochondrial function. Because the elderly are in a basic state of mild inflammation, COVID-19 appears to be particularly fatal. Melatonin can improve the normal physiological functions of the mitochondria and reduce free oxygen radical production. Therefore, we believe that melatonin use in the elderly is a safe and effective adjunct therapy. Melatonin may also play a crucial role in the treatment of novel viral variants. Further, enhancing an individual’s immunity and reducing basal inflammation using melatonin could be a useful strategy against COVID-19.

## Author Contributions

W-LS, M-TL, C-CW, C-LL, and K-CL drafted the manuscript and obtained revision documents. S-FW, M-CL, and C-CW performed data analysis. W-LS, M-TL, C-LL, and K-CL conducted the research. C-CW and W-LS were responsible for collecting examination data. W-LS, K-CL, M-TL, C-LL, and S-FW designed the study. All authors have read and agreed to the published version of the manuscript.

## Conflict of Interest

The authors declare that the research was conducted in the absence of any commercial or financial relationships that could be construed as a potential conflict of interest.

## Publisher’s Note

All claims expressed in this article are solely those of the authors and do not necessarily represent those of their affiliated organizations, or those of the publisher, the editors and the reviewers. Any product that may be evaluated in this article, or claim that may be made by its manufacturer, is not guaranteed or endorsed by the publisher.

## Glossary

ACE, angiotensin-converting enzyme; AGII, angiotensin II; Akt, protein kinase B; AMPK, AMP-activated protein kinase; BAX, Bcl2-associated X; Bcl2, B-cell lymphoma 2; Bcl-xL, Bcl-2-like protein 1; BIM, Bcl2-interacting mediator of cell death; CAT, catalase; cGAS, cytoplasmic ring GMP-AMP synthase; COVID-19, coronavirus disease; DAMP, danger-related molecular pattern; ER, endoplasmic reticulum; ERGIC, endoplasmic Reticulum-Golgi intermediate compartment; ETC, electron transport chain; GSH-Px, glutathione peroxidase; GSH, glutathione; HMGB1, high-mobility group box-1; ICAM, intracellular adhesion molecule; IMP, internal membrane protease; IRF, interferon regulatory factor; MAVS, mitochondrial antiviral-signaling proteins; MDA-5, melanoma differentiation-associated gene 5; Mfn, mitochondrial melting factor; MOM, mitochondrial outer membrane; MPTP, mitochondrial permeability transition pore; mtHSP70, mitochondrial heat shock 70 kDa protein; mTORC1, mechanistic target of rapamycin receptor complex 1; NF-κB, nuclear factor kappa light chain enhancer of activated B cells; NICD, intracellular domain of Notch; NLRP3, NLR family pyrin domain containing 3; Nrf2, nuclear factor erythroid 2-related factor 2; OPA1, optical atrophy 1; PAMPs, pathogen-associated molecular patterns; PRR, pattern recognition receptor; RIG-I, retinoic acid-inducible gene I; RLR, retinoic gene I-analog receptor; ROS, reactive oxygen species; SARS-CoV-2, severe acute respiratory syndrome coronavirus-2; SIRT, sirtuin; SOD, superoxide dismutase; TACE, TNF-α conversion enzyme; TBARS, thiobarbituric acid reaction products; TBK1, TANK-binding kinase 1; TGEV, transmissible gastroenteritis coronavirus; TLR, Toll-like receptor; TNF, tumor necrosis factor; TOM, outer membrane translocase; UPS, ubiquitin-proteasome system; Δψm, mitochondrial membrane potential.
